# Congenital Hepatic Cyst in Patients With Patau Syndrome: A Rare Clinical Finding

**DOI:** 10.7759/cureus.46377

**Published:** 2023-10-02

**Authors:** Andrew C Rennick, Osmay Cardoso, Khushi Saigal, Joseph Boateng, Gaurav Saigal

**Affiliations:** 1 Radiology, University of Miami Miller School of Medicine/Jackson Memorial Hospital, Miami, USA; 2 Radiology, University of Florida College of Medicine, Gainesville, USA; 3 Interventional Radiology, University of Miami/Jackson Memorial Hospital, Miami, USA; 4 Radiology, University of Miami, Miami, USA

**Keywords:** magnetic resonance imaging, abdominal ultrasound, abdominal cyst, congenital hepatic cyst, trisomy 13, patau syndrome

## Abstract

Trisomy 13 (T13), frequently referred to as Patau syndrome, is a rare autosomal aneuploidy most commonly due to nondisjunction in meiosis. Frequently seen characteristics include cleft lip, cleft palate, cerebral defects, anophthalmia, and polydactyly among many more. We report a rare case of a newborn female with T13, demonstrating several known anomalies associated with the syndrome and an associated large congenital hepatic cyst, exhibiting a significant mass effect on vital organs. Based on a literature review conducted in August 2023, we found no previous documentation of a congenital hepatic cyst reported with T13.

## Introduction

Trisomy 13 (T13), also known as Patau syndrome, is a rare chromosomal disorder characterized by the presence of an extra copy of chromosome 13. It is a condition with a grim prognosis, affecting approximately 1 in 12,000 live births, and resulting in a survival rate of less than 10% for affected infants in their first year of life [1]. This syndrome presents a complex clinical picture, encompassing a range of physical abnormalities, observed in our patient [1-6]. However, the presence of congenital hepatic cysts alongside T13 has not been reported in the medical literature until now, making this case an unprecedented occurrence. As such, this case report serves to expand our understanding of the potential comorbidities associated with Patau syndrome and highlights the importance of considering possibly a new clinical manifestation of T13.

## Case presentation

A full-term newborn female was delivered by a 40-year-old mother via cesarean section (C-section). Her prenatal course was significant for an abnormal noninvasive prenatal test (NIPT) that demonstrated concerns for trisomy 13 (T13). On her physical examination, several dysmorphic features were seen including bitemporal narrowing, prominent flat nasal bridge, low set and malformed ears, a sacral dimple, and bilateral postaxial polydactyly.

**Figure 1:**
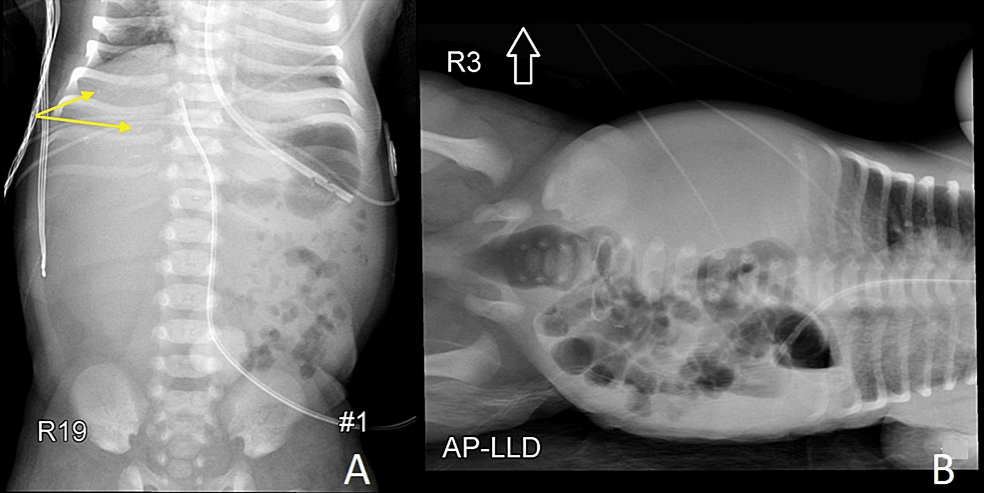
Anteroposterior (A) and left lateral decubitus (B) radiographic views of the abdomen. The images demonstrate absence of gas in the right hemiabdomen and a few small coarse calcifications predominantly in the upper abdomen (yellow arrows; A). There is a mass effect causing deviation of the UVC catheter to the left as well as displacement of the bowel loops to the left. AP-LLD: anteroposterior left lateral decubitus

The patient underwent extensive workup to evaluate for concomitant organ anomalies associated with T13. An abdominal x-ray showed bowel loops localized to the left hemiabdomen and the absence of gas in the right hemiabdomen suggesting an underlying right-sided abdominal pathology. There were also scattered calcifications noted in the upper abdomen, which were originally felt to be due to meconium peritonitis (Figures 1A, 1B).

**Figure 2:**
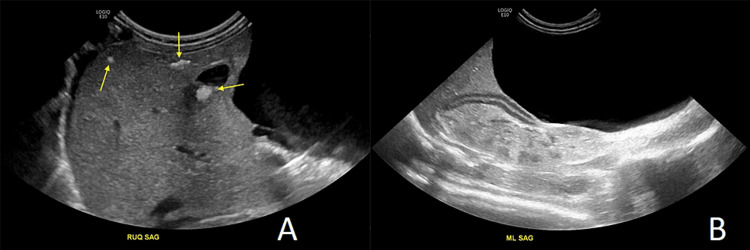
Grayscale sonographic evaluation of the abdomen. The images show multiple hyperechoic foci in the liver (arrows), suggestive of parenchymal calcifications that correspond to those seen on the abdominal x-ray (A). A large anechoic cyst occupying nearly the entire hemiabdomen was also noted (B). RUQ SAG: right upper quadrant sagittal; ML SAG: midline sagittal

Following this, an ultrasound was done which demonstrated an echogenic liver with multiple hyperechoic foci with posterior acoustic shadowing consistent with the calcifications seen on the abdominal x-ray (Figure 2A). A large cyst with a thin wall occupying the right hemiabdomen was also noted which correlated to the absence of gas in the right hemiabdomen seen on the x-ray (Figure 2B).

**Figure 3:**
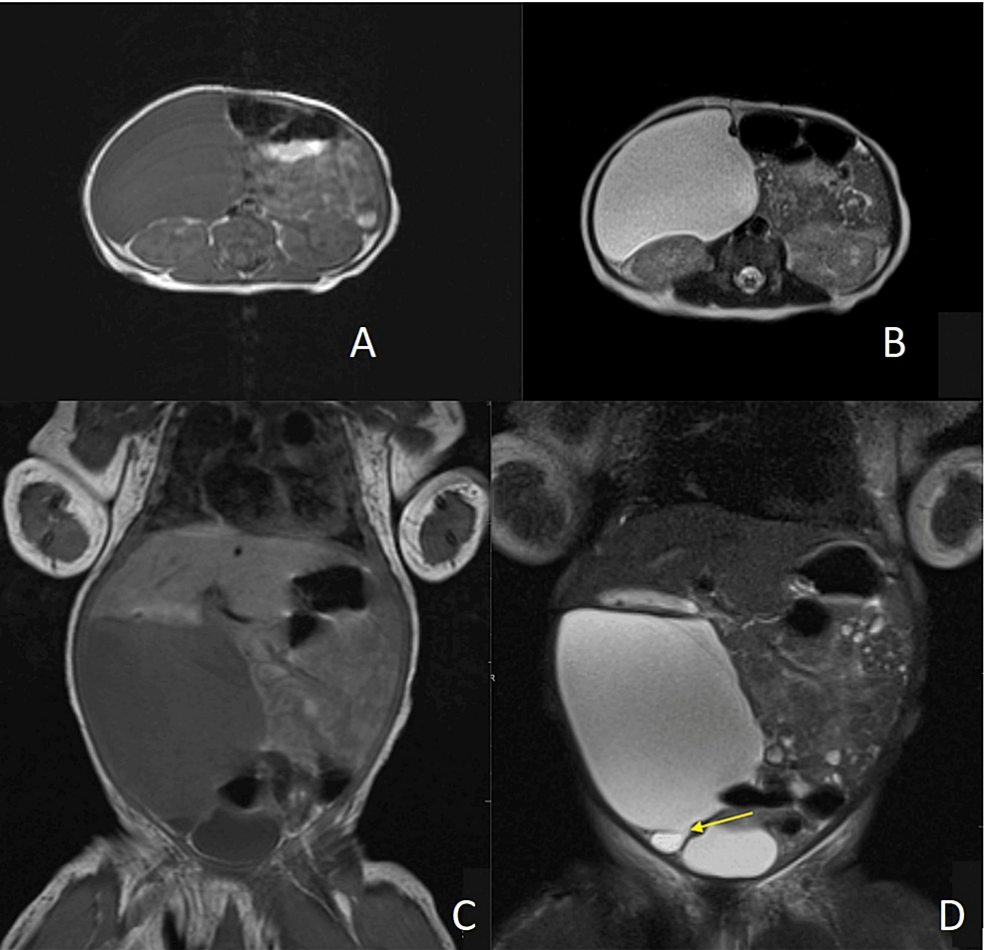
Axial and coronal T1-weighted (A and C) and T2-weighted (B and D) magnetic resonance images of the abdomen. The images demonstrate a large right hemiabdomen hypointense T1/hyperintense T2 mass, compatible with a simple cyst. At the inferior margin of the cyst, the mass was seen to be connected with another cyst, which was felt to be possibly due to an ovarian origin (arrow; D). Significant mass effect on the inferior liver as well as displacement of the bowel loops to the left is noted.

Further workup with magnetic resonance imaging (MRI) of the abdomen with contrast demonstrated a large simple cyst. The cystic structure measured 6.3 x 4.2 x 7.4 cm and occupied the entire right hemiabdomen - extending from the infrahepatic right hemiabdomen to the right pelvis. Significant mass effect was appreciated with asymmetric left hemiabdomen displacement of the bowel and superior displacement of the liver (Figures 3A-3C). Findings were suspicious for a large ovarian cyst given what appeared to be a connection between the cystic mass and the right ovary (Figure 3D).

**Figure 4:**
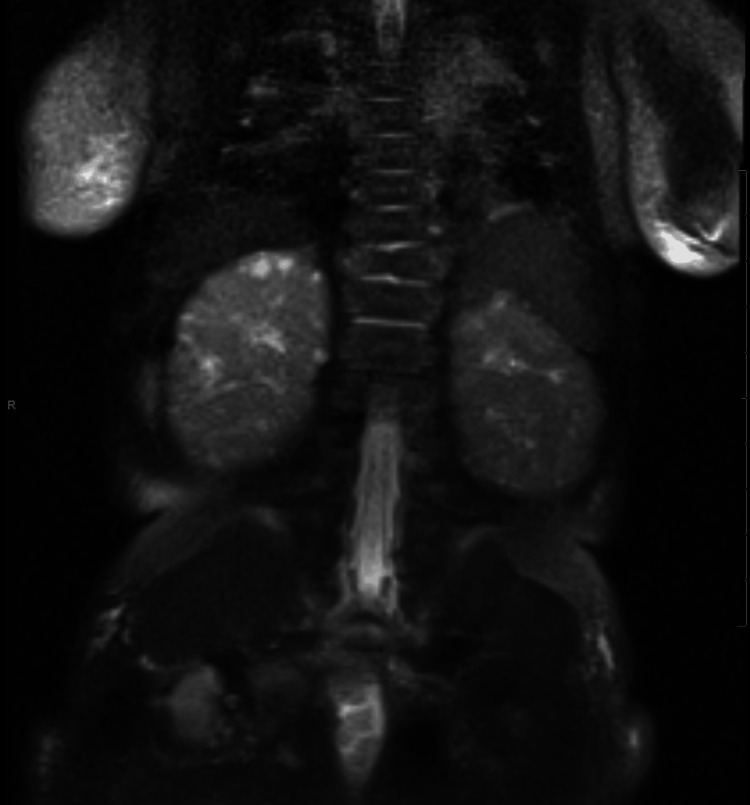
Coronal T2-weighted image of the abdomen. The image demonstrates heterogeneous, dysplastic-appearing kidneys bilaterally with numerous scattered cysts in the renal parenchyma.

Incidentally, heterogeneous kidneys with diffuse tiny parenchymal cysts were observed on MRI, correlating with cystic renal dysplasia commonly described in Patau syndrome (Figure 4).

**Figure 5:**
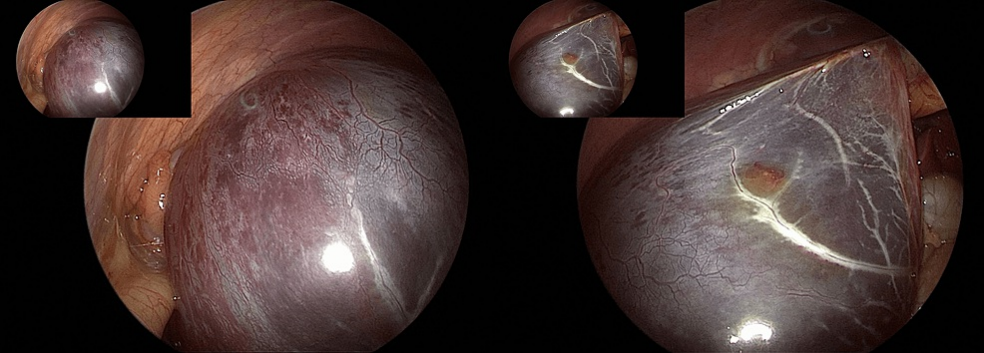
Laparoscopic images acquired during surgical resection. The images demonstrate a large cystic mass originating from the right lobe, centered above the gallbladder. There was no communication between adjacent ovarian cysts or other organs.

On day four of life, the patient began to clinically worsen with increased vomiting concerning for possible malrotation, and she underwent a successful exploratory laparotomy with pediatric surgery. On gross examination, the cyst was arising from the right lobe of the liver, centered above the gallbladder, without connection to other organs (Figure 5). Cystic fluid was clear without evidence of meconium staining or infection. The surgery was uncomplicated and the patient remained hemodynamically stable throughout.

## Discussion

Patau syndrome, also referred to as trisomy 13 (T13), is a rare chromosomal disorder caused by the presence of an extra copy of chromosome 13. Although less common than Down syndrome, T13 is the third most prevalent autosomal trisomy, occurring in approximately 1 in 12,000 live births [1]. The prognosis of Patau syndrome is very poor, with many fetuses dying in utero and less than 10% of infants surviving the first year of life [1].

As seen in our patient, common physical findings of Patau syndrome include bitemporal narrowing, prominent flat nasal bridge, low set and malformed ears, sacral dimple, and bilateral postaxial polydactyly. Findings of intrahepatic calcifications and cystic renal dysplasia have also been associated and well-documented with Patau syndrome [5-9]. Although not seen in our case, Table 1 depicts other common clinical findings among patients with Patau syndrome [1-6]. Congenital hepatic cysts, however, have not been documented in Patau syndrome, and to our knowledge, this is the first reported case of a congenital hepatic cyst in a patient with trisomy 13.

**Table 1:** Summary of clinical features observed in patients with Patau syndrome. *Findings observed in the patient of this case.

Common features associated with Patau syndrome	
Antenatal findings	Polyhydramnios, oligohydramnios, growth restriction
Craniofacial	Abnormal auricles, aplasia cutis, cleft lip, cleft palate, low-set ears*, micrognathia, microphthalmia, ocular hypertelorism, ocular hypotelorism
Congenital heart defects	Atrial septal defect, dextrocardia, patent ductus arteriosus, tetralogy of Fallot, ventricular septal defect
Central nervous system	Holoprosencephaly, microcephaly, ventriculomegaly
Gastrointestinal	Inguinal and umbilical hernias, incomplete colon rotation, Meckel diverticulum, omphalocele
Genitourinary	Cryptorchidism, hypoplasia of labia majora, hypospadias, micropenis
Limbs	Hypoplasia of the nails of hands or feet, postaxial polydactyly*, single palmar crease, rocker bottom feet
Renal	Hydronephrosis, horseshoe kidney, polycystic kidneys*
Skin	Capillary hemangioma, extra skin at the nape

As discussed in this case, the presence of a large cyst in a newborn abdomen can be a diagnostic dilemma. Common etiologies for a large cyst include an ovarian cyst, meconium pseudocyst, choledochal cyst, or an enteric duplication cyst [10,11]. These cysts often appear on ultrasound as anechoic cystic masses that are well-circumscribed and often associated or communicating with the structure of origin [10-12]. Sometimes, in the case of meconium pseudocysts or bleeding, the cyst can be echogenic or complex [12,13]. Given the similarity in presentation, recognizing specific sonographic signs can increase the specificity of the diagnosis. For instance, the visualization of a “daughter cyst” corresponding to an ovarian follicle is 100% specific to ovarian cysts [14]. The “rim” sign of the cyst wall is considered diagnostic of enteric duplication cysts, representing the hypoechoic outer rim of the muscle layer and the echogenic inner rim of the mucosa [10,15]. Choledochal cysts are often described as having a “teardrop” shape, where the proximal end communicating with the biliary tree appears sharp with a round distal end [16]. Understanding these signs can help improve the accuracy of diagnosis; however, if the origin of the cyst cannot be determined via ultrasound, MRI can prove useful in the final diagnosis of lesions [12].

This case report describes another etiology for a large cystic mass in the abdomen - a congenital hepatic cyst. These cysts are rare, with an overall occurrence ranging from 0.1% to 2.5% although recent studies suggest a potentially higher prevalence in the general population [17]. Much like other cysts, sonographic imaging often reveals an anechoic, unilocular fluid-filled space [18]. Surgical intervention is only deemed appropriate in severe presentations, as in our case, where the cyst was causing significant mass effect and compression of vital organs [18].

## Conclusions

In conclusion, the presentation of an abdominal cyst in a newborn can be a diagnostic dilemma with several causes having been described in literature. When associated with other congenital abnormalities, or with a syndrome such as trisomy 13, further challenges can arise in arriving at an accurate diagnosis. Despite the wide breadth of clinical features and findings described with Patau syndrome, congenital hepatic cysts have not been described before in literature. To our knowledge, this is the first case of such an entity being reported in possible association with this syndrome. Further dedicated research investigating the incidence of congenital hepatic cysts and Patau syndrome is indicated prior to establishing this phenomenon as a true association.
